# Beyond motor neurons: peripheral TDP-43 pathology in skeletal muscle and intramuscular nerves in amyotrophic lateral sclerosis

**DOI:** 10.1093/braincomms/fcag241

**Published:** 2026-06-24

**Authors:** Stefania Corti, Claudia Alberti, Linda Ottoboni, Giulia Magni, Delia Gagliardi, Filippo Marcotti, Simona Zanotti, Maurizio Moggio, Giacomo Pietro Comi

**Affiliations:** Dino Ferrari Center, Department of Pathophysiology and Transplantation (DEPT), University of Milan, Milan 20122, Italy; Neuromuscular and Rare Diseases Unit, Department of Neurosciences, Fondazione IRCCS Ca’ Granda Ospedale Maggiore Policlinico, Milan 20122, Italy; Dino Ferrari Center, Department of Pathophysiology and Transplantation (DEPT), University of Milan, Milan 20122, Italy; Dino Ferrari Center, Department of Pathophysiology and Transplantation (DEPT), University of Milan, Milan 20122, Italy; Neurology Unit, Department of Neurosciences, Fondazione IRCCS Ca’ Granda Ospedale Maggiore Policlinico, Milan 20122, Italy; Neurology Unit, Department of Neurosciences, Fondazione IRCCS Ca’ Granda Ospedale Maggiore Policlinico, Milan 20122, Italy; Neurology Unit, Department of Neurosciences, Fondazione IRCCS Ca’ Granda Ospedale Maggiore Policlinico, Milan 20122, Italy; Neurology Unit, Department of Neurosciences, Fondazione IRCCS Ca’ Granda Ospedale Maggiore Policlinico, Milan 20122, Italy; Neuromuscular and Rare Diseases Unit, Department of Neurosciences, Fondazione IRCCS Ca’ Granda Ospedale Maggiore Policlinico, Milan 20122, Italy; Neuromuscular and Rare Diseases Unit, Department of Neurosciences, Fondazione IRCCS Ca’ Granda Ospedale Maggiore Policlinico, Milan 20122, Italy; Dino Ferrari Center, Department of Pathophysiology and Transplantation (DEPT), University of Milan, Milan 20122, Italy; Neurology Unit, Department of Neurosciences, Fondazione IRCCS Ca’ Granda Ospedale Maggiore Policlinico, Milan 20122, Italy

**Keywords:** TDP-43, amyotrophic lateral sclerosis, skeletal muscle, peripheral proteinopathy, diagnostic biomarker

## Abstract

Amyotrophic lateral sclerosis is a progressive neurodegenerative disease characterized by accumulation of the 43-kDa TAR DNA-binding protein (TDP-43). This neuropathological signature has been well documented within the CNS; however, recent findings indicate that the phosphorylated TDP-43 additionally deposits in peripheral tissues, including skeletal muscle and intramuscular nerves. These data warrant a change of view from a neurocentric perspective of amyotrophic lateral sclerosis pathogenesis towards a broader concept of TDP-43 proteinopathy extending both within and beyond the nervous system.

In this review, we focus on current evidence supporting the presence of TDP-43 pathology in amyotrophic lateral sclerosis skeletal muscle, examining its topographic distribution, molecular characteristics and associations with intramuscular nerve bundles. We also discuss the susceptibility of intrinsic muscle cells, disrupted axonal transport and impairment in protein quality control. Phosphorylated TDP-43 pathology in muscle biopsies from amyotrophic lateral sclerosis patients has emerged as a promising tool in the early diagnosis of the disease. Moreover, we discuss the relevance of these findings to amyotrophic lateral sclerosis pathogenesis and potential therapeutic implications.

## Teaser

Corti *et al*. review evidence that phosphorylated TDP-43 accumulates in skeletal muscle and intramuscular nerves of amyotrophic lateral sclerosis patients, discuss proposed pathogenic mechanisms including cell-autonomous and prion-like pathways and highlight the diagnostic and therapeutic potential of peripheral TDP-43 detection as a tissue biomarker.

## Graphical Abstract

**Graphical Abstract:**
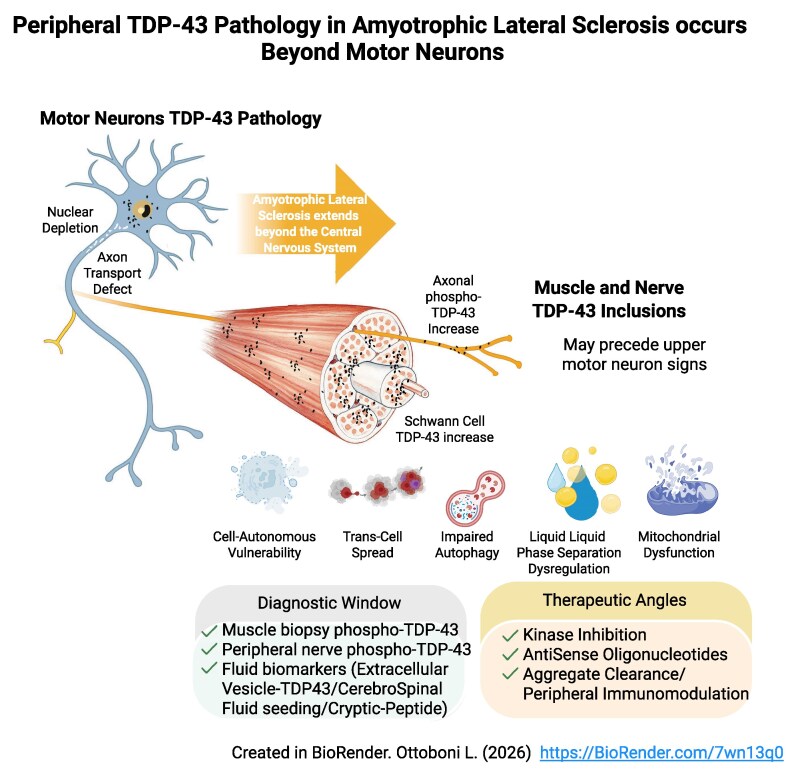

## Introduction

Amyotrophic lateral sclerosis (ALS) is a fatal neurological condition involving progressive motor neuron loss from the brain and spinal cord, leading to muscle weakness, paralysis and ultimately respiratory insufficiency and premature death.^1^ Historically regarded as a disorder confined to motor neurons, ALS is now progressively recognized as a multisystem condition.

For instance, cognitive and behavioural changes, predominantly involving frontotemporal structures, are observed in up to half of ALS patients, while 15% fulfil diagnostic criteria for frontotemporal dementia.^2,3^ Other non-motor symptoms include sensory and autonomic dysfunction and those due to extrapyramidal tract involvement, additionally supporting a conceptual shift in understanding and treating ALS pathophysiology.^4^

Sporadic ALS (sALS) accounts for about 90% of cases, with the remaining 10% being familial (fALS). More than 30 causative genes have now been identified. *C9orf72* hexanucleotide repeat expansions represent the most common genetic cause in European populations, responsible for 40% of fALS and 5–10% of sALS. *SOD1* mutations follow in 12–20% of familial cases, with smaller contributions from *FUS* (4–5%) and *TARDBP* (TDP-43, 3–5% of fALS, <1% of sALS), although these proportions vary across populations.^1,5,6^

More than eighty dominant mutations in *TARDBP* have been associated with ALS, most located in the C-terminal low complexity domain mediating intermolecular protein interactions and liquid–liquid phase separation behaviour. A382T is the most common. Some mutations show striking tissue-specific phenotypes: G376D causes ALS, whereas G376V causes myopathy without neurodegeneration, and the truncation mutation W385IfsX10 leads to rimmed vacuole myopathy rather than classical ALS or frontotemporal dementia. Many of these mutations are aggregate-prone, with some showing increased aggregation through altered post-translational modifications or disulphide bond formation.^6,7^

One of the key neuropathological features of ALS is the aggregation of the TAR DNA-binding protein 43 kDa (TDP-43) in cytoplasmic aggregates. Following the identification of TDP-43 as the principal constituent of ubiquitinated inclusions observed in ALS and frontotemporal lobar degeneration,^8,9^ TDP-43 pathology has been detected in roughly 97% of cases, independent of *TARDBP* mutation status.^10^ The exceptions are *SOD1*- and *FUS*-related ALS, where SOD1 or FUS aggregates predominate. Pathological TDP-43 exhibits hyperphosphorylation and ubiquitination and is susceptible to aggregation, generating cytoplasmic inclusions following nuclear clearance of the protein.

TDP-43 pathology in ALS displays a characteristic anatomical distribution reflecting disease progression. According to the proposed Braak-like staging model, pathology originates in the agranular motor cortex or in brainstem motor nuclei and propagates through anatomically linked regions, ultimately affecting frontal and parietal neocortex, striatum and medial temporal lobe structures, consistent with intercellular propagation.^6^

Although research on TDP-43 pathology has focused mainly on the CNS, evidence has accumulated that TDP-43 deposition is not limited to neurons and glial cells. Phosphorylated TDP-43 (pTDP-43) inclusions have been detected in skeletal muscle fibres and intramuscular nerves, indicating ALS as a systemic TDP-43 proteinopathy.^11,12^

The widespread presence of TDP-43 pathology highlights its central role in ALS pathobiology and its potential as both a therapeutic opportunity and a source for disease biomarkers.

In this review, we will critically synthesize the current evidence for TDP-43 pathology in skeletal muscle in ALS and discuss the proposed mechanisms for peripheral TDP-43 deposits, the relationship and differences between other TDP-43 neuromuscular diseases, along with the potential role of TDP-43 muscle aggregates for the pathogenesis and treatment of ALS.

## TDP-43 structure and function

**FIGURE 1:**
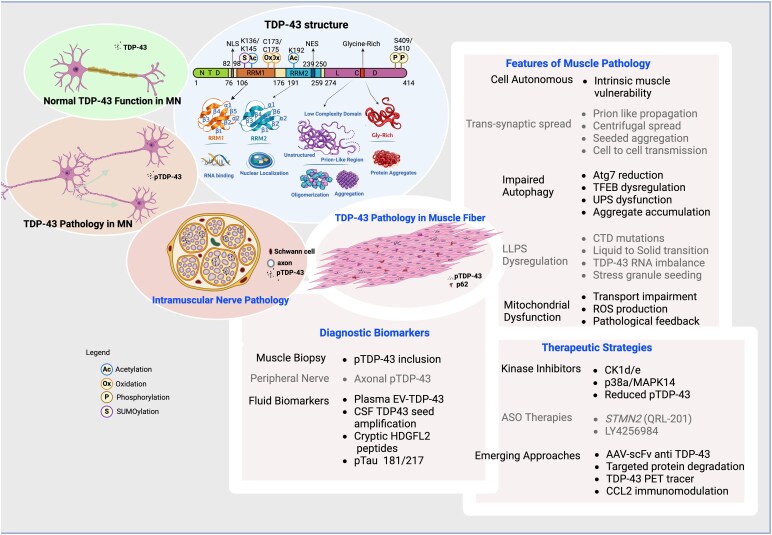
**TDP-43 pathology in skeletal muscle and intramuscular nerves in amyotrophic lateral sclerosis (ALS).** The figure illustrates the distribution and mechanisms of peripheral TDP-43 pathology in ALS, together with its structural basis, diagnostic relevance and therapeutic implications. Under physiological conditions, TDP-43 localizes to motor neuron (MN) nuclei. In ALS, nuclear depletion and cytoplasmic mislocalization yield hyperphosphorylated aggregates (pTDP-43) in motor neuron soma and axons, with proposed centrifugal spread towards skeletal muscle. In muscle fibres, pTDP-43 inclusions colocalize with p62/SQSTM1, indicating autophagic pathway engagement. Five pathogenic mechanisms are depicted: cell-autonomous muscle vulnerability, prion-like trans-synaptic propagation, impaired autophagy, LLPS dysregulation and mitochondrial dysfunction. A cross-sectional schematic illustrates intramuscular nerve involvement, with axonal pTDP-43 detected in 98.2% of ALS patients versus 30.4% of controls, and Schwann cell immunoreactivity in 70.2% versus 17.4% (Riva *et al*., 2022^15^). The central domain map depicts TDP-43 (414 aa), with the NTD (aa 1–76) mediating oligomerization, NLS (aa 82–98) and NES governing nucleocytoplasmic shuttling, RRM1/RRM2 (aa 106–176; 191–259) conferring RNA binding and the glycine-rich CTD (aa 274–414) driving LLPS and aggregation, with major phosphorylation sites at Ser409/410. The schematic also indicates additional post-translational modification sites relevant to TDP-43 pathology, including SUMOylation at K136, acetylation at K145 and K192 and cysteine oxidation at C173 and C175. Diagnostic approaches include muscle biopsy pTDP-43 detection (sensitivity 94.4%, specificity 83.3%; Zhang *et al*., 2024^16^), peripheral nerve pTDP-43 immunoreactivity and fluid biomarkers (plasma EV-TDP-43, CSF seed amplification, cryptic HDGFL2 peptides, P-tau181/217). Therapeutic strategies encompass kinase inhibitors (CK1δ/ε, p38α/MAPK14), ASO therapies (*STMN2*/QRL-201, LY4256984) and emerging approaches (AAV-scFv anti-TDP-43, targeted protein degradation, TDP-43 PET, CCL2 immunomodulation). Created in BioRender. Ottoboni, L. (2026) https://BioRender.com/cl5lu6m. Abbreviations: AAV, adeno-associated virus; Ac, acetylation; ALS, amyotrophic lateral sclerosis; ASO, antisense oligonucleotide; Atg7, autophagy-related 7; CCL2, C-C motif chemokine ligand 2; CK1δ/ε, casein kinase 1 delta/epsilon; CSF, cerebrospinal fluid; CTD, C-terminal domain; EV, extracellular vesicle; HDGFL2, hepatoma-derived growth factor-like 2; LLPS, liquid-liquid phase separation; MAPK14, mitogen-activated protein kinase 14; NES, nuclear export signal; NLS, nuclear localization signal; NTD, N-terminal domain; Ox, oxidation; PET, positron emission tomography; P-tau, phosphorylated tau; pTDP-43, phosphorylated TDP-43; PTM, post-translational modification; ROS, reactive oxygen species; RRM, RNA recognition motif; scFv, single-chain variable fragment; SQSTM1, sequestosome-1; STMN2, stathmin-2; SUMO, small ubiquitin-like modifier; TFEB, transcription factor EB; UPS, ubiquitin-proteasome system.

TDP-43 is a protein consisting of 414 amino acids, encoded by the *TARDBP* gene on chromosome 1. TDP-43 was originally identified as a factor binding TAR DNA elements in the HIV-1 genome, but has since been recognized as a versatile RNA-binding protein implicated in multiple aspects of RNA metabolism.^13^ The TDP-43 protein is structurally divided into four major domains, namely the N-terminal domain (NTD), two RNA recognition motifs (RRMs) and the C-terminal domain (CTD). Though the NTD takes part in homotypic protein interactions and oligomerization, the two RRM domains are the primary RNA-binding modules. The CTD has a glycine-rich region that contains a prion-like domain (PrLD) that is the key element of disease pathogenesis.^14^ Key functional and pathology-associated residues across these domains are illustrated in Fig. 1.^15,16^

The NTD region of the protein affects the equilibrium of the monomeric, dimeric and oligomeric TDP-43 forms, a dynamic process believed to influence the protein's cellular activity.^17,18^ Two major dimerization modes have been reported in literature: head-to-head and head-to-tail, both stabilized by hydrophobic residues and β-strands. Oligomerization occurs through head-to-tail NTD interactions mediated by charge complementarity, salt bridges and hydrogen bonds.^19^

The RRM1 is responsible for guanine-uracil-rich sequence binding in pre-mRNA introns and 3′-untranslated regions (3′ UTRs), whereas the RRM2, although sequence non-specific, can enhance the binding capacity for RNA molecules.^20,21^

The TDP-43 protein autoregulates its expression: when the protein concentration is high, its binding to the TDP-43 binding region in the 3′ UTR of its mRNA becomes more prominent; this results in alternative splicing and the eventual degradation of the resulting unstable RNA molecules.^20^ Pathogenic variants in the 3′UTR can disrupt this autoregulatory feedback loop, leading to protein overexpression and aggregation. These variants represent a distinct gain-of-function mechanism, separate from coding region mutations.^20^

TDP-43 is mainly a nuclear protein and has been implicated in numerous RNA processing functions but under cellular stress, it is rapidly transported to the cytoplasm where it is recruited to stress granules and is involved in mRNA metabolism and translation regulation.^22^ In physiological conditions, this relocalization is not permanent: TDP-43 is restored in the nucleus with the resolution of stress. However, long-term or repeated stress may induce aberrant liquid–solid phase changes, which favour the creation of aggregates that are resistant to dissolution. ALS-related mutations especially those that impact the low-complexity domain facilitate this transition by destabilizing the liquid phase, promoting the transition of dynamic condensates into gel-like or solid aggregates. This process provides a direct connection between stress response in cells and the pathological tendencies of ALS.^6^

## TDP-43 pathology in ALS

The discovery of TDP-43 inclusions in the central nervous system of ALS and frontotemporal lobar degeneration patients, and the subsequent identification of *TARDBP* mutation associations with TDP-43 proteinopathy, offer very strong supporting evidence for the role of the protein in neurodegeneration.^19^ The exact pathomechanisms, however, remain only partially clear. Two different mechanisms have been proposed: the loss-of-function mechanism, in which nuclear depletion impacts the protein's biological role, and the gain-of-function mechanism, which involves the acquisition of toxic properties in cytoplasmic inclusions.^19^

In ALS, the TDP-43 cellular localization undergoes profound changes, typified by nuclear depletion and generation of insoluble, hyperphosphorylated cytoplasmic aggregates within neuronal and glial cells.

Among the biochemical alterations associated with pathological TDP-43 in ALS are hyperphosphorylation, particularly at serines 409 and 410, ubiquitination, cytoplasmic mislocalization with corresponding nuclear reduction and proteolytic fragmentation yielding aggregation-prone C-terminal fragments, most commonly around ∼25 kDa.^19,23^ Although these modifications were initially characterized in central nervous system tissues, there is growing evidence of comparable molecular signatures in skeletal muscle, supporting an extension of TDP-43 proteinopathy beyond the CNS, as discussed in the following sections.

Impaired nucleocytoplasmic transport (NCT) contributes to cytoplasmic TDP-43 accumulation in ALS. The nuclear pore glycoprotein NUP62, a major component of the nuclear pore complex, appears to mislocalize to the cytoplasm and promote TDP-43 insolubility.^24^ In sporadic ALS, TDP-43 mislocalization is accompanied by perturbations of the nuclear import machinery, including altered interactions with karyopherin-dependent pathways, and by increased levels of 14-3-3θ, which binds pathogenic TDP-43 and facilitates its cytoplasmic accumulation.^25^ Finally, cyclophilin A (PPIA) deficiency has been implicated in TDP-43 mislocalization through dysregulation of Ran-dependent nucleocytoplasmic transport, further reinforcing NCT dysfunction as a central pathogenic mechanism in ALS.^26^

## Evidence for TDP-43 pathology in skeletal muscle of ALS patients (Table 1, Figs 1 and 2)

### Early studies

The first studies on TDP-43 alterations in skeletal muscle yielded negative results. Sorarù *et al*.^23^ evaluated quadriceps biopsies from 30 ALS patients and 30 controls by immunohistochemical analysis and Western blot. TDP-43 immunoreactivity was consistently restricted to an intranuclear localization, with no cytoplasmic mislocalization. Moreover, Western blot detected only the typical 43-kDa band without any sign of pathological C-terminal fragments, identical to controls. These data indicated that TDP-43 proteinopathy was confined to the central nervous system in ALS patients. The studies reviewed in this and subsequent subsections included both male and female patients; however, sex-disaggregated analyses of peripheral TDP-43 pathology were not systematically reported across all studies.

**TABLE 1:** Compilation of studies evaluating pTDP-43 pathology in skeletal muscle and peripheral nerves of amyotrophic lateral sclerosis patients

Authors	Study population	Tissue examined	pTDP-43 positivity	Main observations	Diagnostic performance
Sorarù *et al*., 2010^23^	30 ALS patients	Quadriceps muscle (biopsy specimens)	0% (non-phosphorylated isoform)	TDP-43 was detected exclusively in myonuclei; no cytoplasmic aggregates or C-terminal fragments were identified	Not evaluated
Cykowski *et al*., 2018^11^	57 ALS patients (148 muscle samples)	Axial (paraspinal, diaphragm) and appendicular (deltoid, quadriceps) muscles—autopsy	33.3% of patients; 16.2% of samples	Axial muscles showed higher involvement than appendicular; p62 colocalization was observed; no association with CNS TDP-43 burden or C9orf72 expansion	Not evaluated
Mori *et al*., 2019^12^	30 ALS, 13 NMD, 7 non-NMD controls	Tongue, cervical muscles, diaphragm, iliopsoas, heart—autopsy	100% (≥1 region); skeletal: 93%; cardiac: 40%	Two inclusion morphologies were identified: dense filamentous and linear; the diaphragm had the highest frequency; p62-positive; also found in NMD (69%) and controls (43%)	Higher inclusion burden in ALS versus controls
Riva *et al*., 2022^15^	57 ALS, 23 non-ALS	Peripheral motor nerve (biopsy)	Axonal: 98.2%; Schwann cells: 70.2% (in ALS patients)	pTDP-43 detectable in histologically unremarkable nerves; in non-ALS group: axonal 30.4%, Schwann cell 17.4%	Potential for early detection
Kurashige *et al*., 2022^27^	10 ALS, 12 controls (autopsy); 71 biopsy cases	Intramuscular nerve bundles (autopsy and biopsy)	100% (autopsy); 100% predictive (biopsy) in the evaluated cohort	33/33 pTDP-43 + individuals developed ALS; 0/38 pTDP-43− progressed to ALS; pathology preceded UMN involvement	Sensitivity 100%; Specificity 100% (assessable cases)
Zhang *et al*., 2024^16^	18 ALS, 54 non-ALS	Skeletal muscle (biopsy)	94.4%	Non-ALS positivity 29.6% (IBM showed highest density); present in SOD1 and C9orf72 cases; absent in FUS-ALS; muscle changes antedated clinical and EMG findings	Sensitivity 94.4%; Specificity 83.3%

Abbreviations: ALS, amyotrophic lateral sclerosis; CNS, central nervous system; EMG, electromyography; IBM, inclusion body myositis; NMD, neuromuscular disease; pTDP-43, phosphorylated TDP-43; UMN, upper motor neuron.

**FIGURE 2:**
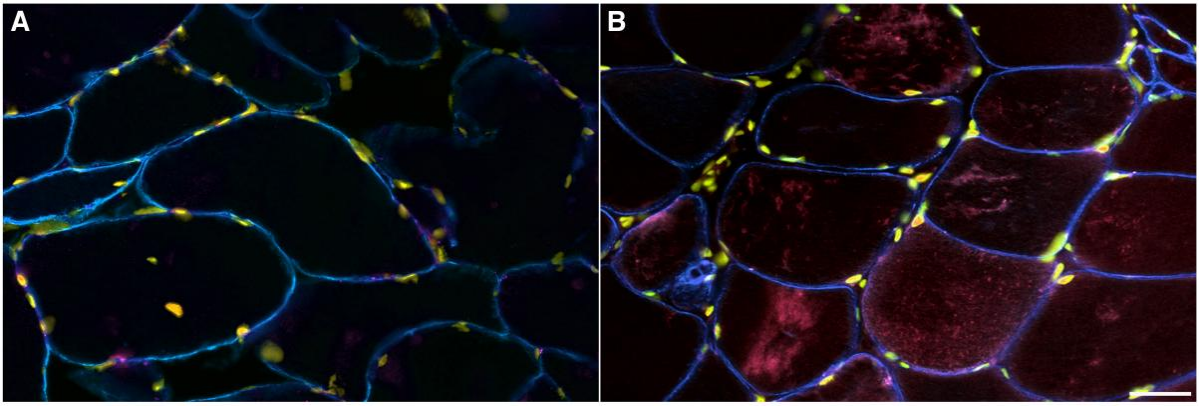
**Immunofluorescence staining of pTDP-43 in skeletal muscle of an ALS patient and a control subject.** Representative immunofluorescence images of skeletal muscle cross-sections from a control subject (**A**) and an ALS patient (**B**). Sections were immunostained for pTDP-43 (Ser409/410; magenta) and laminin (cyan); nuclei were counterstained with DAPI (yellow). Colour-blind-friendly false-colour rendering was applied to enhance accessibility; the original-colour version is provided as Supplementary Fig. 1. (**A**) In control muscle, pTDP-43 immunoreactivity is confined to myonuclei, with no appreciable cytoplasmic signal. Laminin delineates the sarcolemma of normally sized muscle fibres. (**B**) In ALS muscle, several fibres display prominent cytoplasmic pTDP-43 accumulation (magenta) with heterogeneous morphology, intensity and distribution across fibres, including diffuse sarcoplasmic and subsarcolemmal deposits. Fibre size variability is also evident, consistent with denervation changes. Abbreviations: ALS, amyotrophic lateral sclerosis; DAPI, 4′,6-diamidino-2-phenylindole; pTDP-43, phosphorylated TDP-43. Scale bar: 10 μm.

### Recent evidence supporting muscle pTDP-43 pathology

Subsequent studies have challenged these early findings about TDP-43 changes in ALS patients’ muscle tissue. Cykowski *et al*.^11^ (2018) examined 148 autopsy-derived muscle specimens from 57 ALS patients, evaluating both axial (paraspinal and diaphragmatic) and appendicular (deltoid and quadriceps) muscles. The study predominantly included sporadic ALS cases (79%), with familial cases representing 17.5%; *C9orf72* expansion was found in 22.8% of the study population. Cytoplasmic and subsarcolemmal pTDP-43 immunoreactivity was detected in muscle fibres of 19 patients (33.3%) across 24 tissue samples (16.2% of specimens). These inclusions were of varied morphologies, such as blocky, dot, dash and filamentous forms. The appearance of pTDP-43 inclusions in axial muscles was found to occur significantly more frequently compared to appendicular muscles [*P* = 0.0092, odds ratio (OR) = 4.25], with paraspinous muscle accounting for 41.7% and diaphragm for 29.2% of positive samples, while all 26 quadriceps specimens tested negative.

Co-labelling for p62/sequestosome-1 in 23 of 24 pTDP-43-positive samples revealed activation of autophagic pathways, with p62 proving more sensitive than pTDP-43 for detecting inclusion pathology. Real-time quantitative PCR further showed marked upregulation of both *SQSTM1* (4.21-fold, *P* < 0.0001) and *TARDBP* (1.67-fold, *P* = 0.0128) expression in affected muscle tissues. N-terminal TDP-43 staining revealed cytoplasmic aggregates accompanied by loss of normal nuclear immunoreactivity, while FUS immunohistochemistry remained negative. Electron microscopy disclosed subsarcolemmal filamentous material measuring 10–20 nm in width. The distribution of inclusions across multiple fascicles and in both fast- and slow-twitch fibres, along with the lack of correlation between muscle pathology and either disease duration or CNS TDP-43 burden, raises the possibility of cell-autonomous pathogenic processes rather than changes secondary to motor neuron degeneration.

Mori *et al*.^12^, in an autopsy study, investigated samples of five different muscle groups (tongue, cervical muscles, diaphragm, iliopsoas and heart) in 30 ALS patients, 13 patients with other neuromuscular diseases (NMD) and 7 non-NMD controls. When all five muscle regions were systematically examined, pTDP-43 inclusions were detected in at least one region in all 30 ALS patients (100%), with skeletal muscle involvement in 28 cases and cardiac involvement in 12. Of the skeletal muscles, the highest frequency of positivity was found for the diaphragm (19 of 30 cases, 63%), followed by tongue (16 cases), iliopsoas muscle (15 cases) and cervical muscle (6 cases). Statistical analysis confirmed that the mean density of pTDP-43 aggregates in the diaphragm was significantly higher than in cervical muscle and myocardium, pointing to a particular vulnerability of axial respiratory muscles to TDP-43 pathology.

The immunohistochemical analysis identified two morphologically distinct inclusion types: dense filamentous aggregates appearing round or stellate, and short linear filamentous structures. Both types stained positive for native TDP-43 and p62, an autophagy marker, suggesting these aggregates are targeted for autophagic degradation. Proteinase K treatment revealed differential resistance between the two types. Dense filamentous inclusions proved resistant to enzymatic digestion, indicating stronger protein aggregation, while short linear inclusions were sensitive to treatment, reflecting weaker aggregation. Based on these observations, the authors proposed a maturation sequence in which short linear aggregates initially appear diffusely in the cytoplasm, then accumulate in juxtanuclear regions with small vacuoles and progressively evolve into dense filamentous aggregates within dilated vacuoles.

One particularly notable result came from contiguous sections stained with haematoxylin-eosin and anti-pTDP-43. Muscle fibres containing dense filamentous inclusions exhibited single-fibre atrophy with vacuolar degeneration, establishing a direct link between pTDP-43 aggregation and myogenic degeneration rather than purely neurogenic changes. This association was observed in both skeletal and cardiac muscle, representing the first report of pTDP-43 aggregates in myocardium.

Mori *et al*.^12^ further identified pTDP-43 inclusions in 9 of 13 NMD patients (69.2%) and 3 of 7 non-NMD patients (42.9%). NMD-positive cases included a diverse range of disorders such as polymyositis, muscular dystrophies and mitochondrial myopathies. These data indicate that muscle TDP-43 inclusions are not exclusive to ALS but may represent a broader marker of myogenic degeneration across multiple pathological conditions. Nonetheless, the mean density of pTDP-43 aggregates remained significantly elevated in ALS compared to both control groups. The authors suggested that differential autophagy capacity between conditions might account for this variation, positioning ALS as a multisystem proteinopathy that extends beyond the central nervous system into peripheral muscle tissue.

Zhang *et al*.^16^ conducted a diagnostic study performed in a neurogenetic disease centre in China, involving 18 patients with ALS who were chosen from a registry of 802 patients, while the control group consisted of 54 patients who were randomly selected from a muscle biospecimen bank of 698 samples. All the ALS patients had been diagnosed with Airlie House diagnostic criteria and validated among two specialist neurologists, whereas the non-ALS patients were diagnosed with 16 types of neuromuscular disease.

The presence of pTDP-43 deposits was identified in 94.4% (17/18) patients and 29.6% (16/54) non-ALS cases (*P* < 0.001). The deposits also showed a characteristic pattern of subcellular localization, mostly detected around sarcolemmal areas and cytoplasm/perinuclear areas, which were identified by immunofluorescence, immunohistochemistry and electron microscopy. The β-fold conformation of these aggregates was verified using the DANIR 8c fluorescent probe, which specifically recognizes misfolded protein structures.

In the non-ALS group, pTDP-43 was identified in the pathology of several diseases, namely limb girdle muscular dystrophy, inflammatory myopathy (dermatomyositis and polymyositis), neuronal intranuclear inclusion disease (one patient, which is the first reported case in this disease) and inclusion body myositis, with IBM having the highest cumulative density. The study also documented pTDP-43 accumulation in facioscapulohumeral muscular dystrophy, glycogen storage disease, oculopharyngeal muscular dystrophy and oculopharyngodistal myopathy, expanding the known spectrum of conditions associated with this proteinopathy.

As for the genetic subtypes, the pTDP-43 inclusions were detected in *SOD1* mutation patients (*n* = 2) and hexanucleotide repeat expansions of the *C9orf72* gene (*n* = 3), with independent laboratory confirmation of the *SOD1*-ALS findings. On the other hand, the presence of inclusions was not detected in the juvenile ALS patient with *FUS* P525L mutation, in keeping with previous postmortem analysis suggesting that pTDP-43 has diverse roles in the pathogenesis of ALS depending on the underlying genetic cause. Such findings are pertinent to the more general issue of genotype–phenotype correlation regarding TDP-43 pathology. The presence of pTDP-43 in the muscle of *SOD1*-ALS and *C9orf72*-ALS patients establishes that peripheral TDP-43 pathology is not restricted to *TARDBP*-mutated ALS but extends to other genetically heterogeneous subgroups of the disease. In contrast, the absence of pTDP-43 in *FUS*-ALS supports the notion of a separate proteinopathy cascade involving FUS but not TDP-43 aggregates. There are no available data on the comparison of the extent of pTDP-43 in the muscle of patients bearing different subgroups of *TARDBP* mutations. However, as discussed below (see ‘The Super-Aggregation Hypothesis’), the biophysical characteristics of specific *TARDBP* variants are critical in determining tissue tropism: variants that have a higher aggregate-forming potential, like W385IfsX10 and G376V, cause primary myopathies, whereas the very similar G376D mutation leads to neurodegeneration in the absence of primary myopathy. Systematic genotype-muscle pathology correlation studies represent a priority for future investigation.

A semi-quantitative scoring system was used with scores ranging from 0 to 10 for the proportion of pTDP-43-positive cells in at least three randomly chosen fields for a given sample, using a cut-off point of 3 for positivity determined through ROC analysis [AUC 0.910, 95% confidence interval (CI): 0.827–0.993]. The diagnostic test achieved high sensitivity (94.4%) and specificity (83.3%) in distinguishing ALS from other neuromuscular conditions. The average pTDP-43 score was significantly higher in the ALS group than controls (4.7 ± 0.4 versus 0.90 ± 0.2, *P* < 0.001).

Crucially, the muscle pathology in pTDP-43 patients preceded any symptomatology or electromyographic changes in early-stage ALS patients. Seven patients had a single body area affected at the time of biopsy and three patients had myopathic changes rather than neurogenic changes on EMG despite known pTDP-43 protein deposition in muscle tissue. These findings suggest that muscular pTDP-43 deposition may precede neurodegeneration, raising the possibility that skeletal muscle itself could represent a primary site of misfolded protein production rather than merely a downstream target of prion-like spreading from motor neurons.

## TDP-43 accumulations in intramuscular nerves

Riva *et al*.^15^ performed a thorough examination of motor nerve biopsies in 102 patients with lower motor neuron syndromes referred to tertiary neuromuscular centres in Italy. The patient selection was done retrospectively, including those who had undergone obturator nerve biopsy for the anterior motor branch between December 1994 and March 2019, with final clinical diagnoses confirmed after follow-up evaluations indicating the progression of both upper and lower motor neuron abnormalities consistent with ALS or alternative motor neuropathies. Of the 102 patients included, 71 received a final diagnosis of ALS while 31 were classified as non-ALS, the latter group encompassing inflammatory neuropathies, idiopathic neuropathies, myopathies including inclusion body myositis, amyloid polyneuropathy and other conditions.

Differences between ALS and non-ALS subjects were marked for pTDP-43 accumulation. Among the 57 ALS and 23 non-ALS patients with evaluable specimens, axonal pTDP-43 was observed in 98.2% of ALS subjects (56 of 57) versus 30.4% of non-ALS subjects (7 of 23), a highly significant difference (*P* < 0.0001). Cytoplasmic pTDP-43 immunoreactivity in Schwann cells was detectable in 70.2% of ALS subjects (40 of 57) versus 17.4% of non-ALS subjects (4 of 23), also significantly different (*P* < 0.001). Total TDP-43 showed physiologic nuclear staining in all subjects, most prominently in endoneurial, perineurial and vascular nuclei, with no significant difference noted in cytoplasmic TDP-43 staining between ALS and non-ALS subjects. Two independent evaluators blinded to clinical data assessed all biopsies, achieving an inter-reader agreement of 0.96 (Cohen's κ).

Crucially, pTDP-43 pathology was detected in all 11 ALS patients with apparently normal-appearing nerve biopsies based on conventional histopathological analysis, with positive staining for axons in 100% and Schwann cell cytoplasmic inclusions in 54.5%. This indicates that pTDP-43 accumulation in peripheral nerves may represent an early disease event, occurring before the onset of axonal loss detectable through conventional morphological analysis. Motor neurons have a high degree of polarity with axonal projections exceeding the soma volume by several orders of magnitude; thus, peripheral nerve pathology can serve both as a diagnostic biomarker and as a window into disease mechanisms in distal compartments. Axonal transport of RNA granules is disrupted by ALS-linked TDP-43 mutations, potentially depriving neuromuscular junctions of mRNAs essential for local function.

The finding of pTDP-43 in Schwann cell cytoplasm raises the possibility that peripheral glia contribute to disease pathogenesis through non-cell autonomous mechanisms. Schwann cells are essential for axonal homeostasis and repair after injury, and their impairment has been reported in ALS models. Among the non-ALS cases, abnormal pTDP-43 accumulation within myelinated fibres was observed in eight patients (34.8%), including four with idiopathic motor neuropathy, three with myopathy (of which two fulfilled criteria for inclusion body myositis) and one with amyloid neuropathy. These conditions may share pathological mechanisms involving protein aggregation. The research also showed that the rate of axonal degeneration measured in myelin ovoids per square millimetre independently predicted reduced survival in ALS patients when adjusting for age at onset, disease duration and revised El Escorial Criteria category.

Based on their results, Kurashige *et al*.^27^ examined pTDP-43 deposits in nerve bundles of skeletal muscle utilizing a dual postmortem case–control and retrospective cohort design. In the postmortem studies, axonal pTDP-43-immunoreactive inclusions in intramuscular nerve bundles of various muscle types (tongue, diaphragm, biceps brachii and iliopsoas) were seen in all 10 sporadic ALS subjects (mean age at death: 76.1 years; eight men, two women) but not in the 12 non-ALS controls. Immunofluorescence analyses established that these pTDP-43-positive deposits co-localized with axons stained with neurofilament, whereas other pathological protein markers, including FUS, p62 and ubiquitin, were negative in the nerve bundles of ALS patients. Statistical analysis revealed that there was no significant difference in the proportion of affected nerve bundles among the four muscle types investigated which indicates that axonal pTDP-43 deposition is not a localized alteration but rather a common phenomenon in ALS muscle tissue.

The authors also studied muscle biopsy samples of 114 individuals who did not have any previous evidence of neuromuscular disease, selected among 450 consecutive patients who underwent an open muscle biopsy between January 2004 and September 2019. In 71 patients, intramuscular nerve bundles could be assessed, excluding patients with either hereditary or acquired muscle disease, and with a pathogenic variant in known causative genes of ALS. Interestingly, the 33 subjects who had pTDP-43-immunoreactive intramuscular nerve bundles subsequently received the diagnosis of ALS (group A) and the 38 subjects who had no such immunoreactivity did not develop ALS (group B). The remaining 43 patients (group C) did not have any identifiable nerve bundles in the first and subsequent sections; three of them were diagnosed with ALS later.

Of specific diagnostic interest, all 9 subjects with pTDP-43-positive intramuscular nerve bundles who were symptomatic at biopsy had isolated lower motor neuron disease with no upper motor neuron disease, suggesting that axonal pTDP-43 pathology may be the precursor of the clinical syndrome of ALS. Nine patients out of 33 patients who were later diagnosed with ALS had a lower motor neuron isolated presentation at the time of biopsy. Three of these nine had LMN changes in one region only and thus failed to meet the Gold Coast requirements at that point, with the remaining six indicating two or more regions involved. These findings suggest that axonal pTDP-43 pathology can be identified earlier than upper motor neuron abnormalities develop.

The investigation uncovered additional technical considerations relevant to clinical use. In patients with nerve bundles in biopsies, over half (52.1%) of them needed step sections spaced 50-μm apart to detect intramuscular nerve bundles, indicating that comprehensive histological sampling is necessary for reliable detection. The immunohistochemical test with automated staining system of mouse monoclonal and rabbit polyclonal antibody against pTDP-43 (pSer409/410) was better than immunofluorescence in the diagnosis. Patients who presented with both UMN and LMN symptoms at biopsy exhibited shorter time to diagnosis (mean: 4.3 months) than patients who presented with LMN symptoms in one area (8.6 months) or with two or more areas (8.3 months) indicating that pTDP-43 detection may be especially useful in earlier diagnosis of patients with incomplete clinical presentation. Notably, of the 43 cases whose biopsies did not show any recognizable nerve bundles, three of them were subsequently found to have ALS and this shows that negative results should never rule out the diagnosis where nerve bundles cannot be sufficiently evaluated.

## Physiological role of TDP-43 in muscle

The presence of TDP-43 inclusion bodies within skeletal muscle tissue raises the question of the physiological roles of TDP-43 in this tissue. An important proposed role of TDP-43 is the regulation of muscle RNA metabolism, including the regulation of sarcomeric RNAs.^28^ These authors also proposed a role for TDP-43 in skeletal muscle regeneration; they isolated ‘myo-granules’ that were positive for TDP-43 within skeletal muscle following injury and regeneration. Critically, these myo-granules presented amyloid-like properties and were able to seed TDP-43 amyloid fibrils in vitro, suggesting a possible mechanistic link between normal muscle regeneration and pathological TDP-43 aggregation.^28^ As would be expected given a physiological role, lower levels of TDP-43 within *Pax7*-lineage skeletal muscle stem and progenitor cells were shown to impair regeneration and the generation of new muscle fibres.^28^

Further evidence for muscle-specific TDP-43 functions comes from its interactions with non-coding RNAs. TDP-43 interacts with the microRNA-1 (miR-1) family, which promotes differentiation of striated muscle progenitors. It negatively regulates miR-1 activity by limiting its interaction with the RNA-induced silencing complex, thereby modulating the expression of targets such as *IGF-1* and *HDAC4*, the latter being associated with muscle denervation.^29^ TDP-43 also interacts with *Myolinc*, a muscle-specific long non-coding RNA; this interaction facilitates TDP-43 recruitment to the promoter regions of myogenic regulatory factors such as MyoD, underscoring its role in myoblast-to-myotube differentiation.^30^ Tissue-specific differences are also evident in splicing: in muscle, TDP-43 regulates mRNAs encoding proteins involved in DNA-related processes important for differentiation, whereas in neurons, it primarily targets genes related to synaptic activity.^31^

These data suggest that the role of TDP-43 could be muscle-specific and at least in part different from its function in the nervous system. The existence of *TARDBP* mutations causing primary myopathies provides additional support for tissue-specific vulnerability. The loss of muscle-specific TDP-43 function may underlie the skeletal muscle pathology seen in ALS, either as a primary feature or as part of multisystem proteinopathy.

### Mechanisms of TDP-43 aggregation in muscle

Several mechanisms may account for TDP-43 inclusions in ALS muscle tissue (Table 1, Figs 1 and 2).

### Cell-autonomous pathology

One potential interpretation holds that muscle TDP-43 accumulation constitutes a cell-intrinsic process, partially decoupled from motor neuron death. Supporting this interpretation, though not conclusively, is the observation by Cykowski *et al*.^11^ that muscle pTDP-43 pathology did not correlate with overall TDP-43 burden in the central nervous system. The co-localization with p62/SQSTM1, consistently observed across studies,^11,12^ further supports an endogenous proteostasis response within muscle cells.

### Trans-synaptic spread

Alternatively, aggregate formation in muscle tissue might occur by propagation of pathological forms along a motor unit, which would be compatible with a ‘prion-like’ disease propagation model for TDP-43 pathology across the CNS.^32-34^ Although trans-synaptic transfer to myofibers has not been demonstrated, the findings of Kurashige *et al*.^27^, who detected pTDP-43 aggregates in intramuscular nerve bundles, are compatible with a centrifugal orientation of pathology.

Experimental evidence supports this model: pathological TDP-43 seeds extracted from patient tissue can induce aggregation in recipient cells, and brain homogenates containing TDP-43 aggregates induce pathology with spreading to anatomically connected regions in animal models.^6^ Whether such seeded propagation occurs across the neuromuscular junction remains to be directly demonstrated.

Recent evidence supports a bidirectional communication axis between muscle and motor neurons. Muscle-derived extracellular vesicles carrying miR-126a-5p regulate axonal TDP-43 local synthesis; when this signalling is inhibited, TDP-43 accumulates at presynaptic terminals, triggering neuromuscular junction degeneration. Of note, miR-126 introduction exerts neuroprotective effects in both *SOD1*G93A mice and human iPSC-derived co-cultures.^35^

### Secondary response to denervation

Third, muscle TDP-43 deposition may occur secondary to denervation. Denervation leads to a massive reshaping of muscle gene and protein expression, potentially facilitating the mislocalization and aggregation of TDP-43. However, Zhang *et al*.^16^ suggest that denervation alone cannot fully explain muscle pTDP-43 pathology, as pTDP-43 has been detected in muscle tissue sometimes before the onset of clinical manifestations.

### Impaired autophagy

The consistent co-localization of pTDP-43 with p62/sequestosome-1 in muscle cells across multiple studies^11,12,16^ implicates dysfunction of protein quality control pathways. Autophagy abnormalities are well documented in ALS motor neurons and in inclusion body myositis, another TDP-43 proteinopathy,^36^ suggesting that impaired proteostasis may also underlie muscle pTDP-43 inclusions in ALS.

TDP-43 itself regulates autophagy at multiple levels: its depletion reduces *Atg7* mRNA, impairs autophagosome-lysosome fusion through dynactin 1 downregulation and disrupts mTORC1-TFEB signalling.^7^ This creates a potential vicious cycle in which TDP-43 nuclear loss compromises the very clearance pathways needed to remove its cytoplasmic aggregates.

The relative contribution of the UPS versus the autophagy-lysosome pathway to TDP-43 clearance appears context-dependent: the UPS primarily degrades soluble TDP-43, while autophagy becomes critical for clearing aggregated forms and C-terminal fragments. Impairment of either system triggers compensatory upregulation of the other. In muscle, this dual-pathway capacity may confer greater resilience against TDP-43 aggregation compared to neurons.^7^

### Liquid-liquid phase separation

Liquid-liquid phase separation (LLPS) is an additional mechanism by which TDP-43 aggregation can occur in muscle. The C-terminal domain (CTD) contains an intrinsically disordered prion-like domain (PLD), with three tryptophan residues (W334, W385, W412) and a cooperatively formed α-helix (residues 321–330) essential for the process.^37,38^ Although the CTD alone can induce phase separation, NTD-mediated oligomerization enhances it.^39^ A proper TDP-43:RNA ratio is necessary; when cytoplasmic TDP-43 is in excess, homo-oligomerization and inclusion formation ensue.^40,41^ CTD mutations, especially in the α-helix region, disrupt LLPS and promote aggregation.^42^

Cryogenic electron microscopy has revealed that TDP-43 fibrils exist in multiple polymorphic forms with distinct folds across FTLD-TDP subtypes.^43,44^ Structural diversity among fibril conformations may influence tissue-specific vulnerability, although this has not yet been investigated in peripheral deposits.^6^

Each FTLD-TDP subtype shows characteristic clinicopathological associations (type A with behavioural variant FTD; type B with ALS; type C with semantic dementia). Whether distinct fibril conformations also characterize peripheral TDP-43 deposits in muscle remains to be determined.^6^

N-terminal domain fragments may also contribute to TDP-43 pathology: expression of the extreme N-terminus induces ubiquitin-positive inclusions, caspase-3 activation and neurological deficits in transgenic mice.^7^

### Mitochondrial dysfunction

Mitochondrial dysfunction has also been linked to TDP-43 pathology. Overexpression of TDP-43 carrying disease-associated mutations impairs both anterograde and retrograde mitochondrial transport along axons, with mitochondria displaying abnormal morphology, reduced length and accumulation in clusters.^45,46^ TDP-43 binds to mitochondrial DNA transcripts encoding complex I subunits in neuronal cells, and blocking its mitochondrial localization reduces neurotoxicity.^47^ Mitochondrial dysfunction results in an upregulation of reactive oxygen species production, which can further enhance TDP-43 aggregation, potentially initiating a pathological feedback loop.^48^ Whether such pathways are activated in skeletal muscle remains to be determined. Given the high mitochondrial content and oxidative metabolism of skeletal muscle, this pathway may be particularly relevant to muscle TDP-43 pathology. Notably, recent evidence from C2C12 myoblast differentiation experiments has demonstrated that TDP-43 progressively translocates to mitochondria during myotube maturation, paralleling increased expression of myogenic markers.^49^ In skeletal muscle from ALS patients, TDP-43 mitochondrial co-localization is significantly enhanced, particularly within atrophic myofibers. Importantly, pharmacological inhibition of TDP-43 mitochondrial import using a peptide targeting the M1 motif significantly improved myotube fusion efficiency, indicating that aberrant mitochondrial accumulation of TDP-43 directly impairs myogenic differentiation.^49^ These findings provide direct experimental support for a muscle-intrinsic role of mitochondrial TDP-43 in disease pathogenesis and identify TDP-43 mitochondrial mislocalization as a potential therapeutic target in TDP-43 proteinopathies affecting skeletal muscle.

### Additional post-translational modifications

Beyond phosphorylation and ubiquitination, several additional post-translational modifications contribute to TDP-43 pathology. Acetylation of K145 and K192 in the RRMs impairs RNA binding and promotes aggregation.^50,51^ Cysteine oxidation (C173, C175) in RRM1 similarly drives aggregation under oxidative stress.^52^ PARylation modulates LLPS through PAR-binding motifs in the NLS,^53^ while SUMOylation of K136 regulates splicing and nucleocytoplasmic trafficking.^54,55^ Of particular relevance to muscle, the pathological 25 and 35 kDa C-terminal fragments may be degraded more efficiently in muscle cells than in neurons through UPS-autophagy cooperation,^56^ potentially explaining the differential vulnerability of these cell types.

## Comparison with inclusion body myositis and other myopathies

The presence of TDP-43 inclusions in both ALS and inclusion body myositis (IBM) raises several questions regarding the similarities and differences in the underlying pathogenic processes. IBM is an inflammatory myopathy characterized by rimmed vacuoles and multiple protein inclusions, such as β-amyloid, phosphorylated tau and TDP-43. Despite a different clinical presentation, IBM shares a number of pathological similarities with ALS.^36,57^

Notably, TDP-43 pathology has been recapitulated in patient-derived iPSC-myotubes from IBM subjects, which show altered (lower under basal conditions) TDP-43 accumulation together with autophagy dysfunction and mitochondrial alterations, supporting the involvement of TDP-43–linked proteostasis pathways in IBM.^58^ Loss of TDP-43 splicing repression has also been demonstrated in IBM myonuclei, where detection of cryptic HDGFL2 peptides provides a biomarker that correlates with TDP-43 nuclear clearance and supports therapeutic strategies aimed at restoring TDP-43 function.^59^ Finally, TDP-43 aggregates in IBM muscle exhibit seeding capacity in FRET-based biosensor assays: seeding is detected in IBM biopsies but not in immune-mediated necrotizing myopathy or ALS muscle samples, and it anticorrelates with visible aggregate abundance.^60^

Comparative studies have identified similarities and differences between the TDP-43 pathology of ALS and that of IBM. Cykowski *et al*.^11^ established that pTDP-43 inclusion pathology was more extensive and widespread within the muscles of patients with IBM than in those with ALS. Mori *et al*.^12^ confirmed that pTDP-43 inclusion pathology occurs in the skeletal muscles of patients with ALS but also in other conditions related to muscle degeneration. More recently, Zhang *et al*.^16^ confirmed the presence of pTDP-43 inclusion pathology within the muscles of ALS patients versus other conditions, although a direct comparison with IBM was not performed.

TDP-43 inclusions are not exclusive to IBM among myopathies. Similar pathology has been documented in oculopharyngeal muscular dystrophy (OPMD), distal myopathies with rimmed vacuoles (DMRV) and myofibrillar myopathies such as desminopathy and myotilinopathy.^61-63^ More recently, TDP-43 aggregates have been recognized in *HSPB8*-related myopathy, a condition characterized by rimmed vacuoles and stress granule marker accumulation.^64^ The rimmed vacuoles found in sIBM, DMRV and OPMD are in turn associated with autophagy dysfunction.^19^ Collectively, these observations suggest a broader spectrum of TDP-43-related myopathies beyond IBM, potentially sharing common mechanisms of protein quality control dysfunction.

## Novel TDP-43 variants associated with myopathy

Further evidence for TDP-43 pathologies outside the CNS has been offered by recent studies implicating *TARDBP* mutations in primary muscle diseases. In this respect, Ervilha Pereira *et al*.^14^ identified a pathogenic frameshift mutation (p.W385IfsX10) in the prion-like domain of TDP-43 causing rimmed vacuole myopathy, an entity clearly distinct from classical ALS or FTD. More recently, a missense mutation (p.G376V), also located in the prion-like domain, was reported by Zibold *et al*.^65^ to cause late-onset distal myopathy. Both mutations exhibit a marked propensity for forming aggregates, preferentially causing muscle, but not neuronal, degeneration. These data strongly argue against the classical TDP-43 proteinopathy being confined to neurological diseases and suggest potential pleiotropic, tissue-specific properties of *TARDBP* mutations.

## The ‘super-aggregation’ hypothesis

The ‘super-aggregation’ hypothesis has been proposed as a possible explanation for why certain *TARDBP* variants cause primary myopathies rather than classical ALS/FTD. The work of Ervilha Pereira *et al*.^14^ and Zibold *et al*.^65^ suggests that the TDP-43 variants associated with primary myopathies, p.(W385IfsX10) and p.(G376V), have a high propensity for aggregation compared to classical ALS-associated mutations such as p.(M337V) or p.(G376D). Importantly, these highly aggregating TDP-43 variants primarily cause muscle disorders rather than neurodegenerative disorders. These findings are directly relevant to the question of whether specific *TARDBP* mutations cause differential skeletal muscle pathology in ALS (see Evidence section above).

One proposed explanation is that highly aggregating mutant TDP-43 may be sequestered into insoluble inclusions with limited incorporation of wild-type TDP-43, thus potentially protecting against the loss of nuclear function of wild-type TDP-43 in neurons. Because neurons may tolerate partial perturbations of TDP-43 function, these cells might be relatively spared in these conditions. Conversely, skeletal muscle cells may require higher TDP-43 functional thresholds, rendering them more susceptible to haploinsufficiency. This hypothesis challenges the traditional view that protein aggregation is the major contributor to neurotoxicity in ALS.

However, this remains speculative and has been based on observations of relatively few *TARDBP* variants. Further experimental and clinical work will be needed to determine whether super-aggregation constitutes a generally applicable mechanism in TDP-43 proteinopathy.

The identification of myopathy-causing TDP-43 variants places TDP-43 within a growing list of RNA-binding proteins with prion-like domains implicated in primary myopathies, alongside hnRNPA1 and hnRNPA2B1. Mutations in these structurally related proteins share an increased propensity to aggregate, producing muscle inclusions positive for TDP-43. These results reinforce the concept of ‘multisystem proteinopathy’ and point to common pathogenic mechanisms among this protein family.^66^

## Diagnostic implications

The consistent presence of phosphorylated TDP-43 (pTDP-43) inclusions in the muscle tissues of ALS patients reveals the possibility of ALS diagnosis through this avenue. Currently, ALS diagnosis can primarily be made through medical evaluation, electrophysiological studies and the exclusion of other medical conditions exhibiting ALS symptoms, and there is as yet no specific or pathognomonic diagnostic marker for this disease. The estimated diagnostic delay ranges from 10 to 16 months from symptom onset.^67,68^

Zhang *et al*.^16^ demonstrated the diagnostic value of muscle pTDP-43 assessment. Using a semi-quantitative scoring system, the authors achieved high sensitivity (94.4%) and specificity (83.3%) in distinguishing ALS from other conditions. pTDP-43 pathology was detected in patients carrying *SOD1* and *C9orf72* mutations but was notably absent in a *FUS* P525L carrier, consistent with the distinct pathological signature of *FUS*-ALS. From a diagnostic standpoint, muscle changes antedated both clinical manifestations and electromyographic abnormalities in several patients.

However, in view of these promising results, several major issues need to be resolved before the integration of muscle pTDP-43 analysis in the diagnostic workup of sporadic ALS. Detection and quantification techniques require standardization, as differences in immunohistochemical protocols and antibodies may affect diagnostic accuracy. Further validation studies will be necessary to establish more precisely the accuracy of pTDP-43 analysis in appropriately characterized populations of patients with ALS-mimicking disorders. The role of muscle biopsy location is also an important factor, and it appears that axial muscles, including the diaphragm and paraspinal muscles, may be more informative than appendicular muscles. Muscle pTDP-43 analysis needs to be considered a tissue-based pathological marker that needs to be incorporated into, but not supplant, existing diagnostic platforms.

Beyond muscle, skin has emerged as an accessible peripheral tissue for early TDP-43 pathology detection. Using a TDP-43 RNA aptamer combined with *STMN2* cryptic exon probes, Waldron *et al*.^69^ identified pre-symptomatic TDP-43 pathology in skin biopsies from individuals who later developed ALS, detectable up to 26.5 years before motor symptom onset. Sweat and sebaceous glands showed the highest involvement, with shoulder and back representing optimal sampling sites. These results parallel the established utility of skin α-synuclein pathology in Parkinson's disease and suggest that skin TDP-43 detection could serve as an accessible, minimally invasive biomarker for early ALS diagnosis.

The diagnostic landscape for ALS has expanded with recent advances in neurochemical biomarkers.^70^ Quantification of misfolded TDP-43 in plasma extracellular vesicles robustly discriminates ALS from healthy controls, with diagnostic performance showing AUC values >0.9. In subsets of patients with genetically or neuropathologically confirmed TDP-43 proteinopathies, this approach demonstrated high sensitivity and specificity, supporting its potential as a peripheral biomarker of TDP-43 pathology.^71^ Seed amplification assays targeting TDP-43 in CSF and olfactory mucosa samples have also demonstrated high sensitivity for TDP-43 proteinopathies.^72,73^ A novel biomarker reflecting TDP-43 splicing dysfunction, cryptic HDGFL2, shows elevated CSF levels in patients with *C9orf72* ALS/FTD and sporadic ALS, with concentrations peaking in the presymptomatic phase.^74^

Blood phosphorylated tau (P-tau181, P-tau217) has recently emerged as a biomarker linked to lower motor neuron and muscle pathology in ALS. Unlike CSF levels, plasma P-tau is elevated in ALS patients and correlates with LMN signs, faster disease advancement and spinal cord neuronal loss.^75,76^ Immunohistochemical analysis of muscle biopsies reveals P-tau immunoreactivity mainly localized in myonuclei in controls, but in ALS it extends to the sarcoplasm of atrophic fibres, the tunica media of intramuscular vessels and intramuscular axons.^77^ These results suggest that muscle tissue may contribute to elevated blood P-tau levels in ALS, adding further evidence for peripheral pathology in this disease.

## Therapeutic implications

The detection of phosphorylated TDP-43 deposits in muscle from certain ALS patients implies that peripheral TDP-43 alterations may develop via cell-intrinsic mechanisms. This observation carries dual significance: it reveals a possible therapeutic avenue independent of CNS-targeted approaches, and it provides a potential method for tracking treatment efficacy.^11,12^

The modulation of TDP-43 phosphorylation has been explored preclinically. CK1δ/ε phosphorylates TDP-43 at Ser409/410, and its inhibition reduces phosphorylated TDP-43 levels in models.^78-80^ Other kinases implicated in pathological phosphorylation include CK2 (targeting Ser379, Ser403, Ser404), CDC7 (Ser409/410, Ser379), TTBK1/2 and GSK-3. More recently, inhibition of p38α/MAPK14 has been reported to reduce phosphorylation, aggregation, cytoplasmic mislocalization and neurotoxicity in ALS cell models, including motor neurons from ALS patients.^7,81^

Depletion of nuclear TDP-43 causes extensive splicing dysregulation due to aberrant cryptic exon inclusion. TDP-43 negatively regulates cryptic exon inclusion, and its loss results in downregulation of key transcripts, including *STMN2* and *UNC13A*.^82,83^ Loss of *STMN2* causes sensory and motor neuropathy with neuromuscular junction dysfunction, while *UNC13A* loss, crucial for synaptic function, has been associated with symptom onset and cognitive decline in ALS.^84^ TDP-43 also regulates neuronal pentraxin 2 (NPTX2), which is abnormally accumulated in neurons from ALS and FTLD patients with TDP-43 pathology.^6,7,85^

Recent evidence demonstrates that cryptic peptides generated by TDP-43 loss of function can act as neo-antigens recognized by the adaptive immune system. Epitopes derived from HDGFL2 and IGLON5 cryptic peptides are targeted by clonally expanded, highly differentiated CD8+ T cells in ALS and IBM patients. T cells engineered to express these cryptic-specific TCRs can kill TDP-43-deficient astrocytes, providing a direct link between adaptive immunity and TDP-43 pathology.^86^

Antisense oligonucleotides (ASOs) addressing downstream misplicing effects of nuclear TDP-43 loss are currently in development. QRL-201, a splice-modulating ASO to preserve *STMN2* splicing, is under investigation in the ANQUR trial (NCT05633459), while LY4256984 (Eli Lilly, NCT07100119) targets cryptic exon inclusion in *UNC13A*.^87,88^

Preclinical gene therapies have included AAV-mediated approaches expressing targeting antibody fragments like single-chain variable fragment (scFv)s against TDP-43 aimed at suppressing pathologically accumulating and toxic TDP-43.^89^ Targeted protein degradation strategies based on degrader proteins engineered for specific degradation of toxic forms of TDP-43 were also reported in recently published and preprint articles.^89,90^

A promising recent advance is the establishment of [^18^F]ACI-19626, a first-in-class TDP-43 PET tracer now in first-in-human evaluation (NCT06891716). This compound shows high specificity for aggregated TDP-43 and robust binding in FTLD-TDP tissue, although autoradiographic signal in ALS motor cortex appears limited, possibly reflecting lower aggregate density and highlighting the need for *in vivo* validation in ALS cohorts.^91^

Peripheral immunomodulation constitutes a developing therapeutic strategy in ALS. In TDP-43 mouse models, leukocyte and macrophage infiltration occurs in skeletal muscle from presymptomatic stages, preferentially targeting NMJ-enriched regions. Proteomic analysis identified the CCL2-CCR2 chemokine axis as a key driver of this infiltration, with CCL2-expressing cells concentrated around neuromuscular junctions. Local intramuscular administration of CCL2-neutralizing antibodies reduced leukocyte infiltration and ameliorated NMJ denervation, suggesting that targeted peripheral immunomodulation could preserve neuromuscular connectivity in ALS.^92^

Skeletal muscle targeting offers potential advantages, including circumventing the constraints imposed on blood–brain barrier delivery and providing symptomatic effects, although disease-modifying effects remain to be established.

## Limitations and future directions

Although evidence for the presence of TDP-43 pathology in skeletal muscle of ALS patients has been mounting, some important caveats need to be addressed. Many of the studies currently available are limited by small study populations, and it remains challenging to derive conclusions regarding the prevalence and distribution of TDP-43 pathology in skeletal muscle across different ALS variants. The relationship between clinical features and the burden of TDP-43 inclusions in skeletal muscle has not been fully characterized. Furthermore, sex-specific differences in muscle TDP-43 pathology have not been explored; given that ALS prevalence and progression differ between males and females, future studies should incorporate sex as a biological variable in their analyses.

The functional relevance of mislocalization and aggregation of TDP-43 in muscle fibres is also not yet clear. It is not known whether muscle TDP-43 pathology contributes directly to a failure of muscle contractility, metabolism, or regeneration or whether it is only an epiphenomenon of more general disease-related alterations. It is also unclear whether muscle pathology is a primary or secondary process in the context of motor neuron death because the exact timing of central nervous system versus muscle pathology has not been resolved.

To translate current findings into clinical and mechanistic advances, we identify the following research priorities, ranked by translational impact. First, multicentre prospective cohort studies with serial muscle biopsies should be conducted to establish the diagnostic sensitivity, specificity and prognostic value of muscle pTDP-43 across ALS phenotypes, including sex-stratified analyses given known epidemiological differences between male and female patients. Second, *in vivo* mechanistic studies should employ conditional TDP-43 mouse models with muscle-specific (e.g. MCK-Cre) versus neuron-specific (e.g. ChAT-Cre) transgene expression to determine whether muscle TDP-43 accumulation is cell-autonomous or propagated from motor neurons. In vitro human models, including iPSC-derived motor neuron-skeletal muscle co-cultures and neuromuscular organoids will provide complementary platforms to model TDP-43 spreading across the neuromuscular junction. Third, molecular and transcriptomic studies using single-nucleus RNA sequencing and spatial transcriptomics of ALS muscle should define the transcriptomic and splicing consequences of TDP-43 loss-of-function specifically in myonuclei, distinguishing primary myogenic alterations from secondary denervation-related changes. Fourth, standardization of immunohistochemical protocols (including antibody selection, staining conditions and sampling strategies) and development of validated digital pathology scoring systems are essential prerequisites for multicentre diagnostic reproducibility. Fifth, integration of muscle biopsy-based biomarkers with fluid-based markers (plasma EV-TDP-43, CSF seed amplification assays, cryptic HDGFL2 peptides) into multimodal diagnostic frameworks should be pursued in prospective clinical cohorts to determine the optimal combination for early ALS detection. Finally, genotype–phenotype correlation studies systematically comparing the burden of muscle TDP-43 pathology across different ALS-associated genetic backgrounds (*TARDBP*, *SOD1*, *C9orf72*, *FUS*) will help clarify whether the peripheral proteinopathy varies by genetic subtype and whether mutation-specific therapeutic approaches may be warranted.

## Conclusions

Neuropathological studies are increasingly indicating that TDP-43 proteinopathy in ALS is not limited to the central nervous system but also affects the peripheral components of the motor system, including skeletal muscle fibres and intramuscular nerve bundles. A number of studies have now shown that muscle tissue harbours phosphorylated TDP-43 in various subtypes of ALS. This has reinforced the concept that ALS is a multisystem proteinopathy.

Though the pathological importance of peripheral TDP-43 proteinopathy has yet to be fully clarified, its presence has been a constant feature across all studies and its detection in early-stage disease supports both diagnostic and mechanistic relevance. The convergence of tissue-based, fluid-based and imaging biomarkers is beginning to define a peripheral signature of TDP-43 proteinopathy that may complement currently available diagnostic modalities that focus on the central nervous system. Translating these findings into clinical practice will require multicentre validation studies, protocol standardization and mechanistic work to clarify the relationship between peripheral TDP-43 deposits and disease progression. Peripheral TDP-43 pathology represents both a diagnostic opportunity and a potential therapeutic target that warrants systematic investigation.

## Supplementary Material

fcag241_Supplementary_Data

## Data Availability

Data sharing is not applicable. This is a narrative review article; no original data were generated or analysed, and no custom code was developed for this manuscript.
