# Supplementary material for: Beyond motor neurons: peripheral TDP-43 pathology in skeletal muscle and intramuscular nerves in amyotrophic lateral sclerosis
Source: Brain Commun. 2026 Jun 24;8(4):fcag241. doi: 10.1093/braincomms/fcag241 (PMC13332408; doi:10.1093/braincomms/fcag241)
Supplement: fcag241_Supplementary_Data [file fcag241_supplementary_data.pdf]

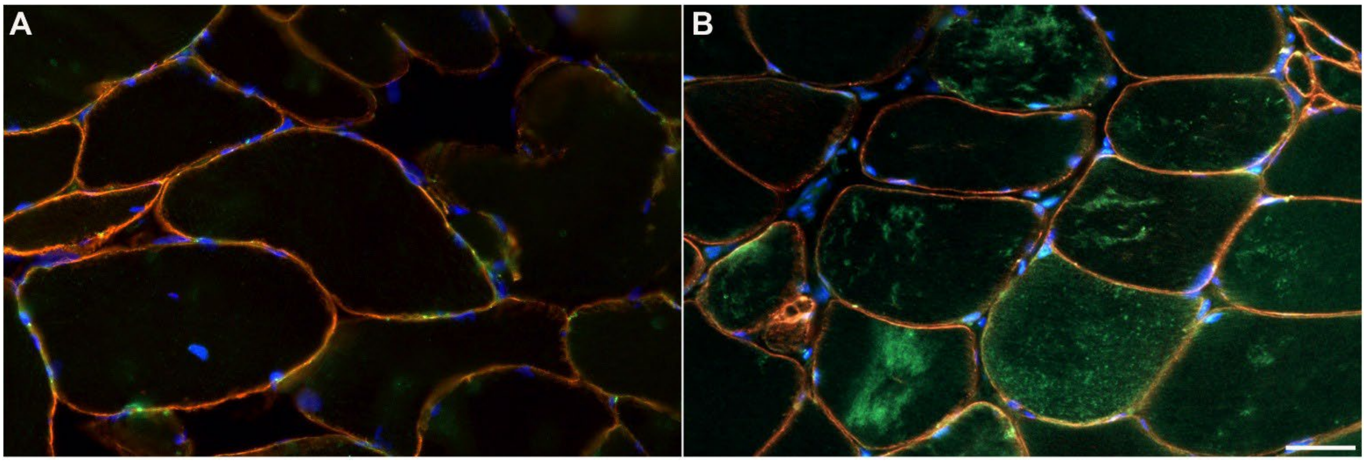

SUPPLEMENTARY FIGURE 1. Original-color immunofluorescence staining of pTDP-43 in skeletal muscle. Same fields as Figure 2, displayed with the original fluorescence channel assignment: pTDP-43 (green), laminin (orange-red), and DAPI nuclear counterstain (blue). **(A)** Control subject. **(B)** ALS patient. This version is provided for reference; the main figure uses a color-blind-friendly false-color palette for accessibility. Scale bar: 10 μm
